# Emotional expression in music: contribution, linearity, and additivity of primary musical cues

**DOI:** 10.3389/fpsyg.2013.00487

**Published:** 2013-07-30

**Authors:** Tuomas Eerola, Anders Friberg, Roberto Bresin

**Affiliations:** ^1^Department of Music, University of JyväskyläJyväskylä, Finland; ^2^Department of Speech, Music, and Hearing, KTH - Royal Institute of TechnologyStockholm, Sweden

**Keywords:** emotion, music cues, factorial design, discrete emotion ratings

## Abstract

The aim of this study is to manipulate musical cues systematically to determine the aspects of music that contribute to emotional expression, and whether these cues operate in additive or interactive fashion, and whether the cue levels can be characterized as linear or non-linear. An optimized factorial design was used with six primary musical cues (mode, tempo, dynamics, articulation, timbre, and register) across four different music examples. Listeners rated 200 musical examples according to four perceived emotional characters (happy, sad, peaceful, and scary). The results exhibited robust effects for all cues and the ranked importance of these was established by multiple regression. The most important cue was mode followed by tempo, register, dynamics, articulation, and timbre, although the ranking varied across the emotions. The second main result suggested that most cue levels contributed to the emotions in a linear fashion, explaining 77–89% of variance in ratings. Quadratic encoding of cues did lead to minor but significant increases of the models (0–8%). Finally, the interactions between the cues were non-existent suggesting that the cues operate mostly in an additive fashion, corroborating recent findings on emotional expression in music (Juslin and Lindström, 2010).

## Introduction

One of the central reasons that music engages the listener so deeply is that it expresses emotion (Juslin and Laukka, 2004). Not only do music composers and performers of music capitalize on the potent emotional effects of music but also the gaming and film industries, as do the marketing and music therapy industries. The way music arouses listeners' emotions has been studied from many different perspectives. One such method involves the use of self-report measures, where listeners note the emotions that they either recognize or actually experience while listening to the music (Zentner and Eerola, 2010). Another method involves the use of physiological and neurological indicators of the emotions aroused when listening to music (recent overview of the field is given in Eerola and Vuoskoski, 2012). Although many extra-musical factors are involved in the induction of emotions (e.g., the context, associations, and individual factors, see Juslin and Västfjäll, 2008), the focus of this paper is on those properties inherent in the music itself which cause emotions to be perceived by the listener that are generally related to mechanism of emotional contagion (Juslin and Västfjäll, 2008).

Scientific experiments since the 1930s have attempted to determine the impact of such individual musical cues in the communication of certain emotions to the listener (Hevner, 1936, 1937). A recent summary of this work can be found in Gabrielsson and Lindström's (2010) study that states that the most potent musical cues, also most frequently studied, are *mode, tempo, dynamics, articulation, timbre*, and *phrasing*. For example, the distinction between happiness and sadness has received considerable attention—these emotions are known to be quite clearly distinguished through cues of *tempo, pitch height*, and *mode*: the expression of happiness is associated with faster tempi, a high-pitch range, and a major rather than minor mode, and these cues are reversed in musical expressions of sadness (Hevner, 1935, 1936; Wedin, 1972; Crowder, 1985; Gerardi and Gerken, 1995; Peretz et al., 1998; Dalla Bella et al., 2001). Other combinations of musical cues have been implicated for different discrete emotions such as anger, fear, and peacefulness (e.g., Bresin and Friberg, 2000; Vieillard et al., 2008).

In real music, it is challenging to assess the exact contribution of individual cues to emotional expression because all cues are utterly intercorrelated. Here, the solution is to independently and systematically manipulate the cues in music by synthesizing variants of a given music. Such a factorial design allows assessment of the causal role of each cue in expressing emotions in music. Previous studies on emotional expression in music using factorial design have often focused on relatively few cues as one has to manipulate each level of the factors separately, and the ensuing exhaustive combinations will quickly amount to an unfeasible total number of trials needed to evaluate the design. Because of this complexity, the existing studies have usually evaluated two or three separate factors using typically two or three discrete levels in each. For example, Dalla Bella et al. (2001) studied the contribution of *tempo* and *mode* to the happiness-sadness continuum. In a similar vein, Ilie and Thompson (2006) explored the contributions of *intensity*, *tempo*, and *pitch height* on three affect dimensions.

Interestingly, the early pioneers of music and emotion research did include a larger number of musical factors in their experiments. For example, Rigg's experiments (1937, 1940a,b, cited in Rigg, 1964) might have only used five musical phrases, but a total of seven cues were manipulated in each of these examples (*tempo, mode, articulation, pitch level, loudness, rhythm patterns*, and *interval content*). He asked listeners to choose between happy and sad emotion categories for each excerpt, as well as further describe the excerpts using precise emotional expressions. His main findings nevertheless indicated that *tempo* and *mode* were the most important cues. Hevner's classic studies (1935, 1937) manipulated six musical cues (*mode, tempo, pitch level, rhythm quality, harmonic complexity*, and *melodic direction*) and she observed that *mode, tempo* and *rhythm* were the determinant cues for emotions in her experiments. Rather contemporary, complex manipulations of musical cues have been carried out by Scherer and Oshinsky (1977), Juslin (1997c), and Juslin and Lindström (2010). Scherer and Oshinsky manipulated seven cues in synthesized sequences (*amplitude variation, pitch level, pitch contour, pitch variation, tempo, envelope*, and *filtration cut-off level*, as well as *tonality* and *rhythm* in their follow-up experiments) but again mostly with only two levels. They were able to account for 53–86% of the listeners' ratings of emotionally relevant semantic differential scales using linear regression. This suggests that a linear combination of the cues is able to account for most of the ratings, although some interactions did occur between the cues. Similar overall conclusions were drawn by Juslin (1997c), when he manipulated synthesized performances of “Nobody Knows The Trouble I've Seen” in terms of five musical cues (*tempo*—three levels, *dynamics*—three levels, *articulation*—two levels, *timbre*—three levels and *tone attacks*—two levels). The listeners rated happiness, sadness, anger, fearfulness, and tenderness on Likert scales. Finally, Juslin and Lindström (2010) carried out the most exhaustive study to date by manipulating a total of eight cues (*pitch, mode, melodic progression, rhythm, tempo, sound level, articulation*, and *timbre*), although seven of the cues were limited to two levels (for instance, tempo had 70 bpm and 175 bpm version). This design yielded 384 stimuli that were rated by 10 listeners for happiness, anger, sadness, tenderness, and fear. The cue contributions were determined by regression analyses. In all, 77–92% of the listener ratings could be predicted with the linear combination of the cues. The interactions between the cues only provided a small (4–7%) increase in predictive accuracy of the models and hence Juslin and Lindström concluded that the “backbone of emotion perception in music is constituted by the main effects of the individual cues, rather than by their interactions” (p. 353).

A challenge to the causal approach (experimental manipulation rather than correlational exploration) is choosing appropriate values for the cue levels. To estimate whether the cue levels operate in a linear fashion, they should also be varied in such a manner. Another significant problem is determining a priori whether the ranges of each cue level are musically appropriate, in the context of all the other cues and musical examples used. Fortunately, a recent study on emotional cues in music (Bresin and Friberg, 2011) established plausible ranges for seven musical cues, and this could be used as a starting point for a systematic factorial study of the cues and emotions. In their study, a synthesis approach was taken, in which participants could simultaneously adjust all seven cues of emotional expression to produce compelling rendition of five emotions (neutral, happy, sad, scary, peaceful, and sad) on four music examples. The results identified the optimal values and ranges for the individual musical cues, which can be directly utilized to establish both a reasonable range of each cue and also an appropriate number of levels so that each of the emotions could be well-represented in at least one position in the cue space for these same music examples.

### Aims and rationale

The general aim of the present study is to corroborate and test the hypotheses on the contribution of musical cues to the expression of emotions in music. The specific aims were: (1) to assess predictions from studies on musical cues regarding the causal relationships between primary cues and expressed emotions; (2) to assess whether the cue levels operate in a linear or non-linear manner; and (3) to test whether cues operate in an additive or interactive fashion. For such aims, a factorial manipulation of the musical cues is required since these the cues are completely intercorrelated in a correlation design. Unfortunately, the full factorial design is especially demanding for such an extensive number of factors and their levels, as it requires a substantial number of trials (the number of factors multiplied by the number of factor levels) and an a priori knowledge of the settings for those factor levels. We already have the answers to the latter in the form of the previous study by Bresin and Friberg (2011). With regard to all the combinations required for such an extensive factorial design, we can reduce the full factorial design by using optimal design principles, in other words, by focusing on the factor main effects and low-order interactions while ignoring the high-order interactions that are confounded in the factor design matrix.

## Materials and methods

A factorial listening experiment was designed in which six primary musical cues (*register, mode, tempo, dynamics, articulation*, and *timbre*) were varied on two to six scalar or nominal levels across four different *music structures*. First, we will go through the details of these musical cues, and then, we will outline the optimal design which was used to create the music stimuli.

### Manipulation of the cues

The six primary musical cues were, with one exception (mode), the same cues that were used in the production study by Bresin and Friberg (2011). Each of these cues has been previously implicated as having a central impact on emotions expressed by music [summary in Gabrielsson and Lindström (2010), and past factorial studies, e.g., Scherer and Oshinsky, 1977; Juslin and Lindström, 2010] and have a direct counterpart in speech expression (see Juslin and Laukka, 2003; except for mode, see Bowling et al., 2012). Five cues—*register, tempo, dynamics, timbre* and *articulation* (the scalar factors)—could be seen as having linear or scalar levels, whereas *mode* (a nominal factor) contains two categories (major and minor). Based on observations from the production study, we chose to represent *register* with six levels, *tempo* and *dynamics* with five levels, and *articulation* with four levels. This meant that certain cues were deemed to need a larger range in order to accommodate different emotional characteristics, while others required less subtle differences between the levels (*articulation* and *timbre*). Finally, we decided to manipulate these factors across different *music structures* derived from a past study to replicate the findings using four different music excerpts, which we treat as an additional seventh factor. Because we assume that the physiological states have led to the configuration of cue codes, we derive predictions for each cue direction for each emotion based on the vocal expression of affect [from Juslin and Scherer (2005), summarized for our primary cues in Table 3]. For mode, which is not featured in speech studies, we draw on the recent cross-cultural findings, which suggest a link between emotional expression in modal music and speech mediated by the relative size of melodic/prosodic intervals (Bowling et al., 2012). The comparisons of our results with those of past studies on musical expression on emotions rely on a summary by Gabrielsson and Lindström (2010) and individual factorial studies (e.g., Scherer and Oshinsky, 1977; Juslin and Lindström, 2010), which present a more or less comparable pattern of results to those obtained in the studies on vocal expression of emotions (Juslin and Laukka, 2003).

### Optimal design of the experiment

A full factorial design with these particular factors would have required 14,400 unique trials to completely exhaust all factor and level couplings (6 × 5 × 5 × 4 × 2 × 3 × 4). As such an experiment is impractically large by any standards, a form of reduction was required. Reduced designs called fractional factorial designs (FFD) and response surface methodologies (RSM), collectively called *optimal designs* provide applicable solutions; however, widespread usage of these techniques within the behavioral sciences is still rare in spite of their recommendation (see McClelland, 1997; Collins et al., 2009). The main advantage of optimal designs over full factorials designs is that they allow the research resources to be concentrated on particular questions, thereby minimizing redundancy and maximizing the statistical power. This is primarily done by eliminating high-order factor interactions (see Myers and Well, 2003, p. 332)^1^.

We constructed the factor design matrix so that the number of cases for each factor level was approximately equal for both main effects and first-order interactions. In this way, the design was compatible with traditional statistical analysis methods and also gave the listener a balanced array of factor combinations. In effect, this meant applying a D-optimal design algorithm to the full factorial matrix, to maximize the determinant of the information matrix (Box and Draper, 1987; Meyer and Nachtsheim, 1995). The number of maximum trials was set to 200, with the intention that each trial would use stimuli with a duration of 25 s, resulting in an estimated 80 min-experiment. The factors are also orthogonal with respect to each other and, thus, are well-suited for statistical techniques such as regression. Details about the individual cues and their levels are given in the next section.

### Details of the seven cues

#### Mode (two nominal levels)

The mode of each music example was altered using a modal translation so that an original piece in an Ionian major scale was altered to the Aeolian minor scale in the same key and vice versa. Thus, the translation from major to minor did not preserve a major dominant chord. For example, the V-I major progression was translated to Vm-Im. This translation was chosen because it allowed a simple automatic translation and also enhanced the minor quality of the examples according to informal listening.

#### Tempo (five scalar levels)

Tempo was represented by the average number of non-simultaneous onsets per second overall voices (called notes per second, NPS). NPS was chosen to indicate tempo because the measure was nearly constant over different music examples when the subjects were asked to perform the same emotional expression in the production study (Bresin and Friberg, 2011). The five different levels were 1.2, 2, 2.8, 4.4, and 6 NPS, corresponding to approximately the median values for the different emotions in the production study.

#### Dynamics (five scalar levels)

The range of the dynamics was chosen corresponding to the typical range of an acoustic instrument, which is about 20 dB (Fletcher and Rossing, 1998). The step size corresponds roughly to the musical dynamics marks pp, p, mp/mf, f, ff: −10, −5, 0, +5, +10 dB, respectively. These values corresponded to the ones obtained in the production study. The dynamics values in dB were controlling the sample synthesizer (see below). The resulting sound was not just a simple scaling of the sound level since also the timber changed according to the input control. This change corresponds to how the sound level and timber change simultaneously according to played dynamics in the real counterpart of the respective acoustic instrument.

#### Articulation (four scalar levels)

The articulation here is defined as the duration of a note relative to its interonset interval. Thus, a value of 1 corresponds to *legato*, and a value of ~0.5, to *staccato*. The articulation was applied using three rules from the previously developed rule system for music performance (Bresin, 2001; Friberg et al., 2006). The *Punctuation* rule finds small melodic fragments and performs the articulation on the last note of each fragment, so it is longer with a micropause after it (Friberg et al., 1998). The *Repetition* rule performs a repetition of the chosen note with a micropause between. Finally, the *Overall articulation* rule simply applies the articulation to all the notes except very short ones. In addition, a limit on the maximum articulation was imposed to ensure that the duration of each note would not be too short. Using this combination of rules, the exact amount of articulation varied depending on the note. However, the four different levels roughly corresponded to the values 1, 0.75, 0.5, 0.25—thus, a range from *legato* to *staccatissimo*. The same combination of rules was used in the production study.

#### Timbre (three scalar levels)

Three different instrument timbers were used for the melody voice: flute, horn, and trumpet. The same timbers were also used in the production experiment and were initially chosen for their varied expressive character, namely brightness, which has been found to have a large impact on emotional ratings in a previous experiment (Eerola et al., 2012). The estimation of brightness was based on the amount of spectral energy below a cut-off of 1500 Hz, because this correlated strongly (*r* = −0.74, *p* < 0.001, *N* = 110) with the listeners' ratings when they were asked to judge the emotional valence of 110 isolated instruments sounds (Eerola et al., 2012). Flute has the lowest and the trumpet has the highest brightness value.

#### Register (six scalar levels)

The whole piece was transposed so that the average pitches of the melody were the following: F_3_, B_3_, F_4_, B_4_, F_5_, and B_5_ corresponding to the MIDI note numbers 53, 59, 65, 71, 77, and 83, respectively. These values were close to the actual settings for the different emotions in the production study.

#### Music structure (four nominal levels)

Finally, the seventh cue music structure was added in order to extend the design across four different music examples chosen from the Montreal battery of composed emotion examples (Vieillard et al., 2008). Each example represented a different emotion and was selected according to how it had been validated by Vieillard et al. (2008). Therefore, the selected examples were from among the most unambiguous examples of sadness (T01.mid in the original stimulus set), happiness (G04.mid), peacefulness (A02.mid), and fear (P02.mid) from the study by Vieillard et al. Because the study consisted of four different musical examples many compositional factors like melody, harmony, and rhythm varied simultaneously; these same four music examples were also used in the previous production study (Bresin and Friberg, 2011).

### Creation of the stimuli

The stimulus examples were generated with an algorithm using the Director Musices software (Friberg et al., 2000). The resulting MIDI files were rendered into sound using the Vienna Symphonic Library with the Kontakt 2 sampler. This library contains high-quality, performed sounds for different instruments using different sound levels, registers, and playing techniques^2^. All the accompaniment voices were played on a sampled piano (Steinway light) and the melody voices were played on samples of each solo instrument (horn, flute, and trumpet). The sound level of each instrument was measured for a range of different MIDI velocity values and an interpolation curve was defined, making it possible to specify the dynamics in decibels, which was then translated to the right velocity value in the MIDI file. The onset delays were adjusted aurally for each solo instrument in such a manner that simultaneous notes in the piano and in the solo instrument were perceptually occurring at the same time. The resulting audio was saved in non-compressed stereo files (16-bit wav) with the sampling rate at 44.1 kHz. Examples of the stimuli are available as Supplementary material (Audio files 1–4 that represent prototypical examples of each rated emotion).

### Procedure

The subjects were sitting either in a semi-anechoic room (Stockholm) or in a small laboratory room (Jyväskylä). Two loudspeakers (Audio-Pro 4–14 in Stockholm/Genelec 8030 in Jyväskylä) were placed slightly behind and either side of the computer screen. The sound level at the listening position was calibrated to be at 72 dB (C). Several long notes of the horn were used as the calibration signal, performed at the middle scalar value of *dynamics* (0 dB—as detailed above).

The subjects were first asked to read the written instructions (in Swedish, English, or Finnish). Their task was to rate each example (*n* = 200) on each of the emotions provided (four concurrent ratings for each example). They were asked to focus on emotional expression (i.e., perceived emotions rather than felt emotional experiences) of the example and the ratings were made on a seven-point Likert scale. The emotions were tender/peaceful, happy, sad, angry/scary in Stockholm and tender, peaceful, happy, sad, and angry in Jyväskylä. One reason behind the variation in terms between the laboratories was to compare the terms used in the original study by Vieillard et al. (2008) to terms frequently used by other studies adopting the basic emotion concepts for music (e.g., Bresin and Friberg, 2000; Juslin, 2000; Juslin and Lindström, 2010; Eerola and Vuoskoski, 2011). The second reason to vary the labels was to explore whether collapsing the ratings of similar emotions (e.g., tender and peaceful) would result in large differences when compared to the uncollapsed versions of the same emotions. A free response box was also provided for the participants to use in cases where none of the given emotion labels could be satisfactorily used to describe the stimulus. However, we will not carry out a systematic analysis of these textual responses here, as they were relatively rare (the median number of excerpts commented on was 2 out of 200, the mean 3.4, *SD* = 4.7) and the participants that did comment did not comment on the same examples, which further hinders such an analysis.

The stimuli were presented in a different random order for each participant. The scale's position had no influence on response patterns. The experiment itself was run using the program Skatta^3^ at Stockholm and a patch in MAX/MSP at Jyväskylä. For each example, there was a play button and four different sliders for the corresponding emotion labels. The subject was free to repeat the examples as many times as he/she wished. The whole session took between 1 and 2 h to complete. The subjects were also encouraged to take frequent pauses, and refreshments were available.

### Participants

In all, 46 participants took part in the experiment, 20 in Stockholm and 26 in Jyväskylä. Because the ratings collected in these two laboratories were nearly identical (detailed later), we will not document all the data gathered in each of the laboratories separately. The mean age of all participants was 30.2 years (*SD* = 8.7), 20 of the participants were female and 25 were male; one participant did not indicate his/her gender. Most of the participants had an extensive musical background as, between them, they reported having music as a hobby for an average of 16.1 years (*SD* = 10.5) and studying music at a professional level for an average of 7.0 years (*SD* = 6.3). Their musical taste was a mixture of many styles, and the participants also represented various ethnicities (some of whom were not native speakers of Swedish or Finnish). All participants were compensated for their efforts (≈9 €).

## Results

The description of the analysis will proceed according to the following plan. First, the consistencies of the ratings across and between the emotions will be reported. Next, the main hypotheses will be investigated using a series of regression analyses. The first regression analysis will address the contribution of cues to the emotions, the second one will address the linearity of the cue levels, and the third one will-seek to quantify the degree of interactions between the cues in the data, and compare the results with results obtained using models that are additive. All of the analyses will be carried out separately for each of the four emotions.

### Inter-rater consistency

There was no missing data, and no univariate (in terms of the z-scores) or bivariate outliers were identified (using squared Mahalanobis distances with *p* < 0.05 according to the Wilks' method, 1963). The inter-rater consistency among the participants was high at both laboratories, (the Cronbach α scores were between 0.92 and 0.96 in Stockholm, and 0.94 and 0.97 in Jyväskylä). Because of substantial inter-participant agreement for each emotion, and because individual differences were not of interest, the analyses that follow treat the stimulus (*N* = 200) as the experimental unit, with the dependent variable being the mean rating averaged across all participants. The Pearson correlations between the mean ratings from the two laboratories were also high for the identical emotion labels (*r*_[198]_ = 0.94 and 0.89 for happy and sad, both with *p* < 0.0001 for both). For the emotion labels that were varied between the laboratories, significant correlations between the variants also existed; tender/peaceful (Stockholm) and peaceful (Jyväskylä) correlated highly (*r* = 0.81, *p* < 0.0001, *N* = 200) so did tender/peaceful (Stockholm) and tender (Jyväskylä), *r* = 0.89. In addition, angry/scary (Stockholm) and angry (Jyväskylä) exhibited a similar, highly linear trend (*r* = 0.96, *p* < 0.0001, *N* = 200). Due to these high correspondences between the data obtained from the two laboratories, pooling tender/peaceful (Stockholm) with tender and peaceful (Jyväskylä) to *peaceful*, and, angry/scary (Stockholm) with angry (Jyväskylä) to *scary* was carried out.

### Correlations between perceived emotions

Next, we explored intercorrelations between the emotion ratings by looking specifically at correlations between the four consistently rated emotions (*happy, sad, peaceful*, and *scary*). These displayed a typical pattern, wherein happy correlated negatively with *sad* (*r* = −0.79 p < 0.001 and *N* = 200), and *happy* correlated positively with *peaceful*, albeit weakly (*r* = 0.21, *p* < 0.01), and *happy* correlated significantly with *scary* (*r* = −0.56, *p* < 0.001). *Sad* was weakly correlated with *peaceful* (*r* = 0.16, *p* < 0.05) while *sad* showed no correlation with *scary* (*r* = 0.04, *p* = 0.55). Finally, *peaceful-scary* exhibited significant opposite trend as would perhaps be expected (*r* = −0.72, *p* < 0.001). Similar patterns have also been observed in a study by Eerola and Vuoskoski (2011).

**Figure 1:**
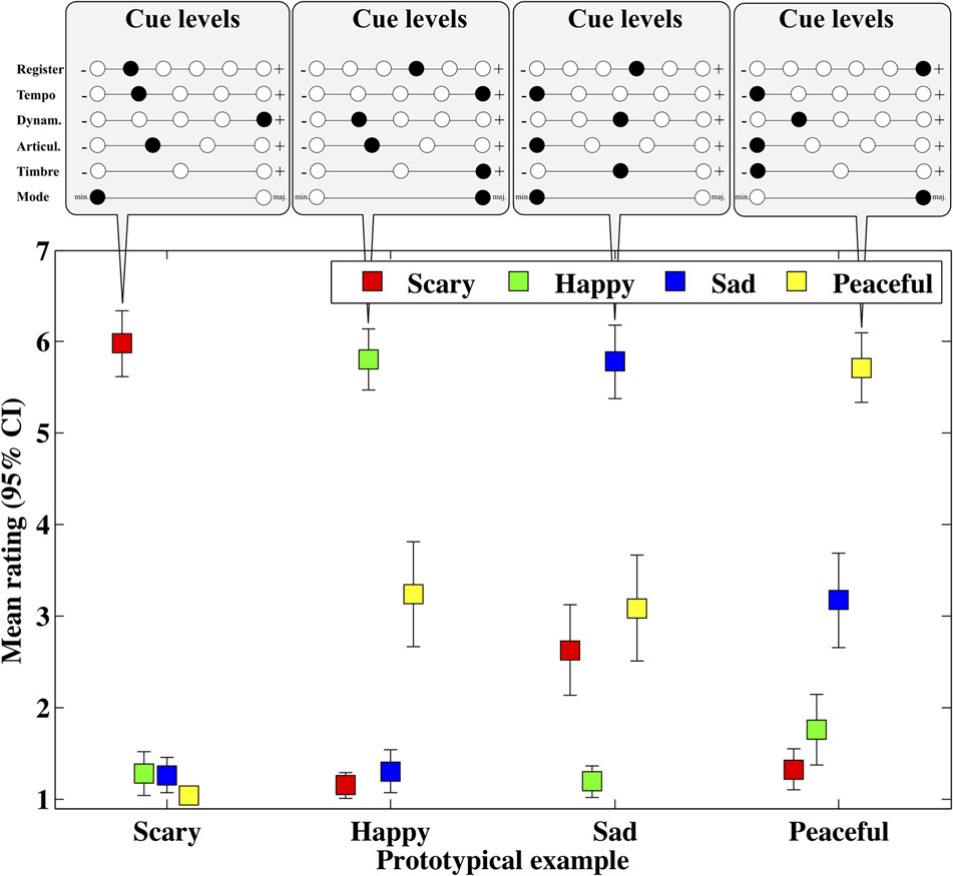
**Means and 95% confidence intervals of four emotion ratings for four prototype examples that received the highest mean on each emotions**.

Next, we investigated the emotion scales with examples that were judged highest for each emotion to see the overall discrimination of the scales (see Figure 1, these examples are also given as audio files 1–4). Each of these prototype examples is clearly separated from the other emotions, yet the overall pattern reveals how particular emotions are related to other emotions. For instance, happy and sad prototypes get modest ratings also in peaceful, and the peaceful prototype scores similar ratings in sadness. However, these overlaps do not imply explicit confusions between the emotions, as evidenced by 95% confidence intervals. This suggests that all four scales are measuring distinct aspects of emotions in this material. The exact cue levels—shown on the top panels—for each prototype, clear show four distinct cue patterns. Interestingly, there are not only extreme cue levels used in the optimal profiles (e.g., low tempo, dynamics, and articulation and high register for peaceful) but also intermediate levels being used (e.g., middle register and dynamics for sad and happy prototypes). However, a structured analysis of the cue contributions is carried out in the next sections.

### Cue contributions to emotions

**Figure 2:**
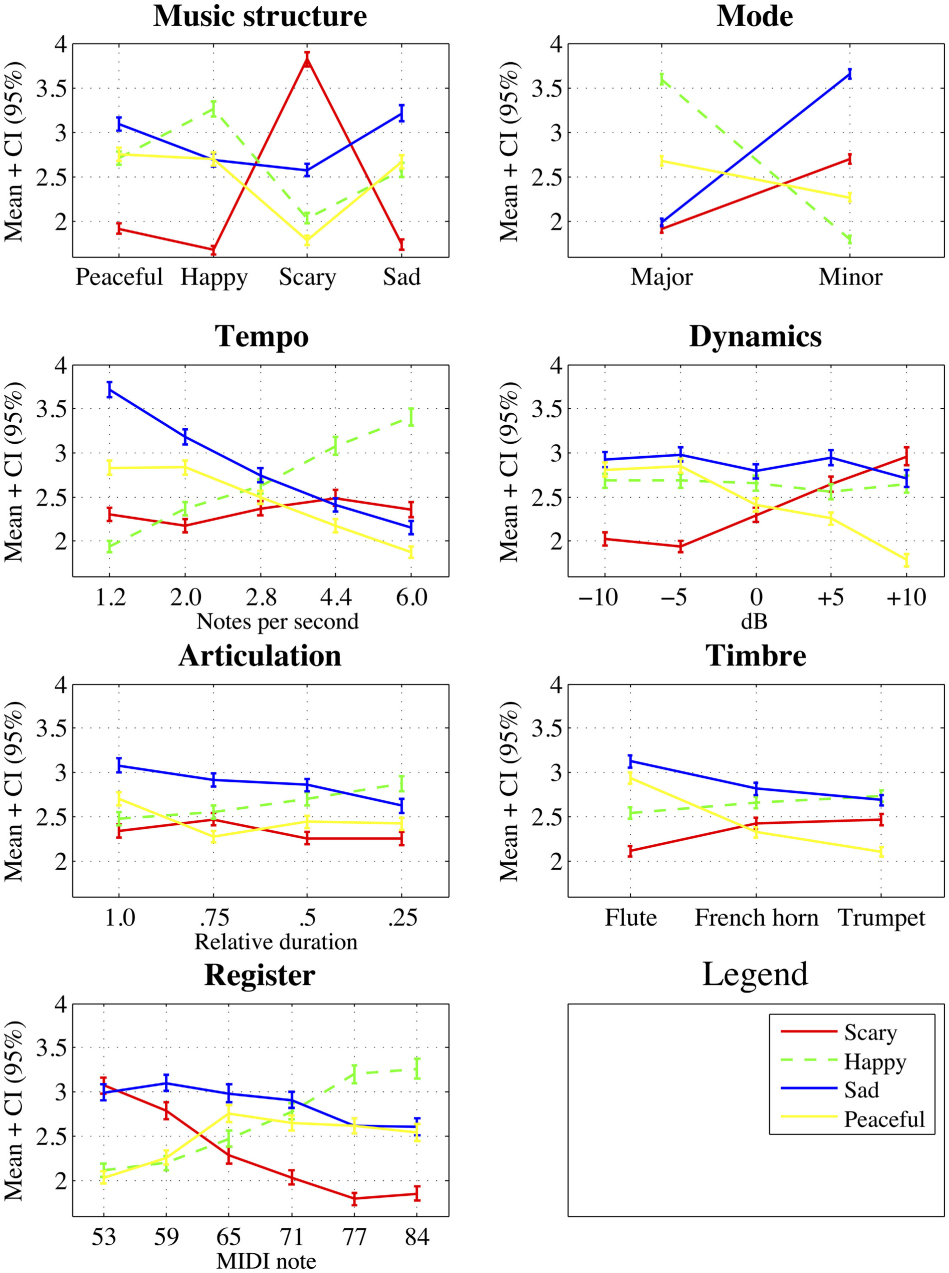
**Means and 95% confidence intervals of four emotion ratings across all musical cues and levels**.

For an overview of the cues and their levels for each emotion rating, a visualization of the mean ratings is given in Figure 2. Most cues exhibited a strikingly clear pattern across the levels for most of the four emotions. For example, *register* can be seen to have had a clear effect on the emotions happiness and fearfulness. A higher register corresponded to a higher happiness rating while a lower register corresponded to a higher fearfulness rating. Similar trends were displayed in *tempo, mode, dynamics* and *articulation*, though the specific emotions and the directions of the cues levels were different. It is also worth noting that the nominal cues, *mode* and *music structure*, showed large differences across the cue levels. This suggests that these cues had a powerful impact on each emotion rating scale. For *music structure*, the appropriate emotion can always be seen as a peak in the mean ratings of that emotion. In other words the prototypically “happy” musical example was consistently rated by participants to be the highest in happiness, not in other emotions. This effect was most pronounced in the case of scary and least evident in peacefulness.

To assess the impact of each cue for each emotion, regression analyses were carried out for each emotion using all the cues (see Table 1).

**Table 1:** **Summary of regression models for each emotion with linear predictors (mode and music structure are encoded in a non-linear fashion)**.

	**Scary**		**Happy**		**Sad**		**Peaceful**		**Median *sr*^2^**
	***R*^2adj^ = 0.85**		***R*^2adj^ = 0.89**		***R*^2adj^ = 0.89**		***R*^2adj^ = 0.77**		
	β	***sr*^2^**	β	***sr*^2^**	β	***sr*^2^**	β	***sr*^2^**	
Mode	−0.74^***^	0.08	1.77^***^	0.48	−1.6^***^	0.54	0.43^***^	0.05	0.29
Tempo	0.07^**^	0.01	0.25^***^	0.12	−0.32^***^	0.21	−0.27^***^	0.15	0.14
Music struct. 3	1.56^***^	0.33	−0.53^***^	0.02	−0.49^***^	0.04	−0.99^***^	0.12	0.08
Register	−0.23^***^	0.15	0.18^***^	0.09	−0.05^*^	0.01	0.15^***^	0.06	0.08
Dynamics	0.20^***^	0.08	−0.01	0.00	−0.05^**^	0.01	−0.28^***^	0.14	0.04
Articulation	−0.03	0.00	0.14^***^	0.02	−0.18^***^	0.04	−0.10^**^	0.01	0.02
Timbre	0.15^***^	0.02	0.01	0.00	−0.14^***^	0.01	−0.45^***^	0.13	0.01
Music struct. 2	−0.18^*^	0.00	0.42^***^	0.03	0.42^***^	0.02	0.02	0.00	0.01
Music struct. 1	−0.11	0.00	0.14	0.00	−0.11	0.00	0.10	0.00	0.00

df = 9,190,

* p < 0.05,

** p < 0.01,

*** p < 0.001. β, standardized betas; R^2adj^, R^2^ adjusted; corrected for multiple independent variables.

As can be observed from the Table 1, the ratings of all emotions can be predicted to a high degree (77–89%) by a linear coding of the five scalar cues. Beta coefficients facilitate the interpretation of the model and the squared semipartial correlations (*sr*^2^) are useful for showing the importance of any particular cue within the regression equation as it shows the unique proportion of variance explained by that cue. The cues are ranked along the median *sr*^2^ values across the emotions. Note that the *music structure* cue is displayed using three dummy-coded variables, allowing us to discriminate between the effects related to the four different music structures used. Scary is predominantly communicated by the structure of the music (a nominal cue), in that a combination of low register, minor mode, and high dynamics contributes to these ratings. The most effective way of expressing happiness is a major, fast tempo, high register, and staccato articulation within this particular set of examples. For sadness, the pattern of beta coefficients is almost the reverse of this, except a darker timber and a decrease in dynamics also contributes to the ratings. These patterns are intuitively clear, consistent with previous studies (Juslin, 1997c; Juslin and Lindström, 2003, 2010).

The first thing we see is that the relative contributions of the cues vary markedly for each emotion, just as in previous studies (Scherer and Oshinsky, 1977; Juslin, 1997c, 2000; Juslin and Lindström, 2010). For example, *mode* is extremely important for happy and sad emotions (*sr*^2^ = 0.48 and 0.54), whereas it has a relatively low impact on scary and peaceful (*sr*^2^ = 0.08 and 0.05). Similar asymmetries are apparent in other cues as well. For instance, *dynamics* significantly contributes to scary and peaceful emotions (*sr*^2^ = 0.08 and 0.14) but has little impact on happy and sad (*sr*^2^ = −0.01 and 0.01). This latter observation is somewhat puzzling, as previously, dynamics has often been coupled with changes in valence (Ilie and Thompson, 2006) and happy or sad emotions (Adachi and Trehub, 1998; Juslin and Laukka, 2003). However, when direct comparisons are made with other factorial studies of emotional expression (Scherer and Oshinsky, 1977; Juslin, 1997c; Juslin and Lindström, 2010), it becomes clear that dynamics have also played a relatively weak role in sad and happy emotions in these studies. If we look at the cues that contributed the most to the ratings of sadness, namely *mode* and *tempo*, we can simply infer that the ratings were primarily driven by these two factors.

The overall results of the experiment show that the musical manipulations of all cues lead to a consistent variation in emotional evaluations and that the importance of the musical cues bears a semblance to the synthetic manipulations of musical cues made in previous studies. We will summarize these connections later in more detail. Instead of drawing premature conclusions on the importance of particular musical cues and the exceptions to the theory, we should wait until the specific properties of the cue levels have been taken into account. These issues will therefore be addressed in-depth in the next section.

### Linearity versus non-linearity of cue levels

We used hierarchical regression analysis to estimate three qualities of the cue levels (namely linear, quadratic, and cubic) as well as the overall contribution of the cue themselves because this is the appropriate analysis technique for an optimal design with a partial factor interaction structure (e.g., Myers and Well, 2003, pp. 615–621; Rosenthal and Rosnow, 2008, p. 476).

The cue levels were represented using (a) linear, (b) quadratic and (b) cubic using the mean ratings over subjects (200 observations for each emotion). Each emotion was analyzed separately. This was applied to all five scalar cues. For completeness, the nominal cues (*mode* and *music structure*) were also included in the analysis and were coded using dummy variables.

*Mode* used one dummy variable, where 0 indicated a minor and 1 a major key; while music structure used three dummy variables in order to accommodate the non-linear nature of the cue levels. None of the cues were collinear (variance inflation factors <2 for all cues) as they were the by-product of optimal factorial design. Table 1 displays the prediction rates, the standardized beta coefficients as well as squared semi-partial correlations for each cue and emotion.

The Step 1 of the hierarchical regression is equal to the results reported in Table 1. Based on Figure 2 and previous studies, we might think that linear coding does not do full justice to certain cues, such as *register* or *timbre*. To explore this, we add quadratic encoding of the five cues (register, tempo, dynamics, articulation, and timbre) to this regression model at Step 2. As quadratic encoding alone would reflect both linear and quadratic effects, the original linear version of the variable in question was kept in the analysis to partial out linear effects (Myers and Well, 2003, pp. 598–559). Adding the quadratic variables at the Step 2 results in increased fit for scary [+3%, *F*_(185, 5)_ = 10.0, *p* < 0.001], sad [+0.05%, *F*_(185, 5)_ = 2.4, *p* < 0.05], and peaceful [+8%, *F*_(185, 5)_ = 23.5, *p* < 0.001] emotions but no increase for the ratings of happy emotion (see Table 2). For the ratings of scary emotion, quadratic versions of *register, dynamics*, and *timbre* are responsible for the increased fit of the model which suggests that these particular cues do contribute to the emotions in non-linear fashion.

**Table 2:** **Hierarchical regression comparing linear, quadratic, and cubic predictors**.

	**Scary**			**Happy**		**Sad**		**Peaceful**	
	***df***	***R*^2adj^**	***F*∆**	***R*^2adj^**	***F*∆**	***R*^2adj^**	***F*∆**	***R*^2adj^**	***F*∆**
Step 1. Linear	9,190	0.85		0.89		0.89		0.77	
Step 2. Quadratic	5,185	0.88	10.0^***^	0.89	0.68	0.89	2.4^*^	0.85	23.9^***^
Register^2^			^***^		–		–		^***^
Tempo^2^			–		–	^**^	^***^
Dynamics^2^			^***^		–		–		^***^
Articulation^2^			–		–		–		^**^
Timbre^2^			^***^		–		–		^***^
Step 3. Cubic	5,180	0.88	0.75	0.89	0.97	0.89	1.3	0.86	1.8
Register^3^			–		–		–		–
Tempo^3^			–		–		–		–
Dynamics^3^			–		–		–		–
Articulation^3^			–		–		–		–
Timbre^3^			–		–		–		–

df refers to number of predictors in the model, F∆ denotes the comparison of model at Steps 1, 2, and 3 for the complete regression models, and also the individual significance (t) of the cues,

*** p < 0.001,

** p < 0.01,

* p < 0.05.

A similar observation was made in the ratings of peacefulness. A quadratic variant of the timbre, register, tempo, articulation, and dynamics provided statistically significant change to model at Step 2 (+8.0%, see Table 2). Ratings of Sad emotion also received a marginal, albeit statistically significant, change at Step 2 due to contribution of quadratic encoding of *tempo*. The overall improvement of these enhancements will be presented at the end of this section. At Step 3, cubic versions of the five cues (register, tempo, dynamics, articulation, and timbre) were added to the regression model but these did not led to any significant improvements beyond the Step 2 in any emotion (see Table 2).

For all of these cues and emotions, cubic variants of the cue levels did not yield a better fit with the data than with quadratic versions. It is also noteworthy that the quadratic versions of the cues were included as additional cues, in that they did not replace the linear versions of the cues. It suggests that some of the cue levels violated the linearity of the factor levels. Therefore, small but significant quadratic effects could be observed in the data mainly for the cues of *timbre, dynamics* and *register*, and these were specifically concerned with the emotions of scary and peacefulness. In the context of all of the cues and emotions, the overall contribution of these non-linear variants was modest at the best (0–8% of added prediction rate) but nevertheless revealed that linearity cannot always be supported. Whether this observation relates to the chosen cue levels or to the actual nature of cues, remains open at present. The overarching conclusion is that the many cue levels were successfully chosen and represented linear steps based on the production experiment (Bresin and Friberg, 2011). These selected levels predominantly communicated changes in emotional characteristics to the listeners in a linear fashion.

### Additivity vs. interactivity of the cues

Previous findings on the additivity or interactivity of musical cues are inconsistent. According to Juslin (1997c); Juslin and Lindström (2010), and Scherer and Oshinsky (1977), cue interactions are of minor importance (though not inconsequential), whereas others have stressed the importance of cue interactions (Hevner, 1936; Rigg, 1964; Schellenberg et al., 2000; Juslin and Lindström, 2003; Lindström, 2003, 2006; Webster and Weir, 2005). To evaluate the degree of cue interactions in the present data, a final set of regression analyses were carried out. In these analyses, each two-way interaction is tested separately for each emotion (21 tests for each emotion) using the mean ratings (*N* = 200). This analysis failed to uncover any interactions between the cues in any emotion after correcting for multiple testing (all 84 comparisons result in non-significant interactions, *p* > 0.315, *df* = 0196). It must be noted that some of the interactions that would be significant without corrections for multiple testing (*register* and *mode*, and *mode* and *tempo* in Happiness, *mode* and *tempo* in Sadness), are classic interacting cues of musical expression (Scherer and Oshinsky, 1977; Dalla Bella et al., 2001; Webster and Weir, 2005), and could be subjected to a more thorough, multi-level modeling with individual (non-averaged) data.

In conclusion, the results of the analysis of additivity vs. interactivity were found to be consistent with the observations made by Scherer and Oshinsky (1977); Juslin (1997c), and Juslin and Lindström (2010) that the cue interactions are comparatively small or non-existent, and additivity is a parsimonious way to explain the emotional effects of these musical cues.

## Discussion

The present study has continued and extended the tradition of manipulating important musical cues in a systematic fashion to evaluate, in detail, what aspects of music contribute to emotional expression. The main results brought out the ranked importance of the cues by regression analyses (cf. Table 1). The nominal cue, *mode*, was ranked as being of the highest importance, with the other cues ranked afterwards in order of importance as follows: *tempo, register, dynamics, articulation*, and *timbre*, although the ranking varied across the four emotions and music structures. Seventy nine percent of the cue directions for each emotion were in line with physiological state theory (Scherer, 1986), and simultaneously, in accordance with the previous results from studies on the cue directions in music (e.g., Hevner, 1936; Juslin, 1997c; Gabrielsson and Lindström, 2001; Juslin and Lindström, 2010). The second main result suggested that most cue levels contributed to the emotions in a linear fashion, explaining 77–89% of variance in the emotion ratings. Quadratic encoding of three cues (*timbre,register*, and *dynamics*) did lead to minor yet significant increases of the models (0–8%). Finally, no significant interactions between the cues were found suggesting that the cues operate in an additive fashion.

A plausible theoretical account of how these particular cue combinations communicate emotional expressions connects the cues to underlying physiological states. This idea, first proposed by Spencer in 1857, builds on the observation that different emotions cause physiological changes that alter vocal expression (e.g., increased adrenalin production in a frightened state tightens the vocal cords, producing a high-pitched voice). This physiological state explanation (Scherer, 1986) is typically invoked to explain emotions expressed in speech, since it accounts for the cross-cultural communication of emotions (Scherer et al., 2001) and assumes that these state-cue combinations have been adapted to common communicational use, even without the necessary underlying physiological states (e.g., Bachorowski et al., 2001). This theoretical framework has an impact on musically communicated emotions as well, because many of the cues (speech rate, mean *F*_0_, voice quality) that contribute to vocally expressed emotions have been observed to operate in an analogous fashion in music (e.g., Juslin and Laukka, 2003; Bowling et al., 2012). This theory enables direct predictions of the cue properties (importance and cue directions) that convey particular emotions. We have compiled the predictions from expressive vocal cues (Juslin and Scherer, 2005) and expressed emotions in music to the Table 3. When we look at the summary of the cue directions from the present study, also inserted to the Table 3, out of 24 predictions of cue directions based on vocal expression, 19 operated in the manner predicted by the physiological state theory (Scherer, 1986), three against the predictions, and two were inconclusive (see Tables 1, 3). Two aberrations in the theory were related to register, which is known to have varying predictions in vocal expression with respect to the type of anger (hot vs. cold anger, see Scherer, 2003). The third conflict with the theory concerns tempo. Previous studies of the musical expression of emotions have suggested *tempo* as the most important cue Gundlach, 1935; Hevner, 1937; Rigg, 1964; Scherer and Oshinsky, 1977; Juslin and Lindström, 2010 and here *mode* takes the lead. We speculate that the nominal nature of *mode* led to higher effect sizes than linearly spaced levels of *tempo*, but this obviously warrants further research.

**Table 3:** **Theoretical prediction and results for each cue and emotion**.

	**Levels**	**Scary Pred./Res.**	**Happy Pred./Res.**	**Sad Pred./Res.**	**Peaceful Pred./Res.**
Register	6 L	Ʌ/˅	Ʌ/Ʌ	˅/˅	˅/Ʌ
Tempo	5 L	Ʌ/=	Ʌ/Ʌ	˅/˅	˅/˅
Dynamics	5 L	Ʌ/Ʌ	Ʌ/−	˅/˅	˅/˅
Articulation	4 L	Ʌ/−	Ʌ/Ʌ	˅/˅	˅/˅
Timbre	3 L	Ʌ/Ʌ	=/=	˅/˅	˅/˅
Mode	2 C	▲/∆	▼/□	▲/∆	▼/∇
Music struct.	4 C	F>H>S>P/ F>S>H>P	H>P>S>F/ H>P>S>F	S>P>H>F/ S>P>H>F	P>S>H>F/ P>S>H>F

L, Linear; C, Categorical; Pred, predictions; Res, results; Ʌ, high; =, moderate; ˅, low; ▲, minor; ▼, major—refers to not statistically significant, and letters in music structure refer to predictions based on Montreal Battery (F, Fearful; H, Happy; S, Sad; P, Peaceful). Predictions are based on Juslin and Scherer (2005), except Mode is based on Gabrielsson and Lindström (2010).

We interpret these results to strengthen that the musical cues may have been adopted from the vocal expression (Bowling et al., 2012 for a similar argument). We also acknowledge the past empirical findings of the expressive properties of music [e.g., as summarized in Gabrielsson and Lindström (2010)] but since these largely overlap with the cues in vocal expression (Juslin and Laukka, 2003), we rely on vocal expression for the theoretical framework and use past empirical studies of music as supporting evidence. It must be noted that expressive speech has also been used as a source of cues that are normally deemed solely musical, such as mode (Curtis and Bharucha, 2010; Bowling et al., 2012).

A further challenge related to the reliable communication of emotions via cue combinations is that the same cue levels may have different contributions to different emotions (e.g., the physiological state of heightened arousal causes a high speech rate or musical tempo, which is the same cue for both fearfulness and happiness, or, as in the reverse situation, a low *F*_0_ conveys boredom, sadness, and peacefulness). An elegant theoretical solution is provided by the Brunswik's lens model (adapted to vocal emotions by Scherer in 1978), which details the process of communication from (a) the affective state expressed, (b) acoustic cues, (c) the perceptual judgments of the cues and (d) the integration of the cues. The lens model postulates that cues operate in a *probabilistic* fashion to stabilize the noise inherent in the communication (individual differences, contextual effects, environmental noise—the same cues may contribute to more than one emotion). Specifically, Brunswik coined the term *vicarious functioning* (1956, pp. 17–20) to describe how individual cues may be substituted by other cues in order to tolerate the noise in the communication. This *probabilistic functionalism* helps to form stable relationships between the emotion and the interpretation. In emotions expressed by music, Juslin has employed the lens model as a framework to clarify the way expressed emotions are communicated from performer to listener (Juslin, 1997a,b,c, 2000).

The cue substitution property of the lens model presumes that there are no significantly large interactions between the cues, because the substitution principle typically assumes an additive function for the cues (Stewart, 2001). Therefore, our third research question asked whether the cues in music contribute to emotions in an additive or interactive fashion. Significant interactions would hamper the substitution possibilities of the lens model. Empirical evidence on this question of expressed emotions in music is divided; some studies have found significant interactions (Hevner, 1935; Rigg, 1964; Schellenberg et al., 2000; Gabrielsson and Lindström, 2001, p. 243; Lindström, 2003, 2006; Webster and Weir, 2005) between the cues when the contribution of 3–5 cues of music have been studied, while other studifes have failed to find substantial interactions in similar designs with a large amount of cues (Scherer and Oshinsky, 1977; Juslin and Lindström, 2010). In the vocal expression of emotions, the importance of the interactions between the cues has typically been downplayed (Ladd et al., 1985). Our second research question probed whether the cues contribute to emotions in a linear fashion. Previous studies have predominantly explored cues with two levels e.g., high-low (Scherer and Oshinsky, 1977; Juslin and Lindström, 2010), which do not permit to draw inferences about the exact manner (linear or non-linear) in which cue values contribute to given emotions (Stewart, 2001). Based on the physiological state explanation, we predicted a high degree of linearity within the levels of the cues, because the indicators of the underlying physiological states (corrugator muscle, skin-conductance level, startle response magnitude, heart rate) are characterized by linear changes with respect to emotions and their intensities (e.g., Mauss and Robinson, 2009). The results confirmed both linearity and additivity of the cue contributions although non-linear effects were significant for some cues.

The most cue levels represented in scalar steps did indeed contribute to emotion ratings in a linear fashion. The exceptions concerned mainly *timbre*, for which we had only three levels. These levels were determined using the single timbral characteristic of *brightness*, but the three instrument sounds used also possessed differences in other timbral characteristics. Nevertheless, the observed relationship between emotions and *timbre* was consistent with previous studies. However, the results of one particular observation proved the hypotheses drawn from the past research wrong. *Dynamics* turned out to be of low importance both for the sad and happy emotions although it has previously been implicated as important for emotions in a number of studies using both emotion categories (Scherer and Oshinsky, 1977; Juslin, 1997c; Juslin and Madison, 1999; Juslin and Lindström, 2010) and emotion dimensions (Ilie and Thompson, 2006). It is unlikely that our results are due to insufficient differences in dynamics (±5 and ±10 dB) because ratings for the emotions peaceful and scary were nevertheless both heavily influenced by these changes. However, they might be related to the specific emotions, as this musical cue has been previously noted to be a source of discrepancy between speech and music (Juslin and Laukka, 2003). Our results are further vindicated by the fact that the emotions happy and sad have not exhibited large differences in dynamics in previous production studies (Juslin, 1997b, 2000).

Finally, the assumption inherent in the lens model that cues operate in additive fashion was validated. The interactions failed to reach statistical significance consistent with comments made by previous surveys of emotional cues (Gabrielsson and Lindström, 2001, p. 243; Juslin and Laukka, 2004) and a number of studies (e.g., Juslin, 1997c; Juslin and Lindström, 2003). This means it should therefore be realistic to construct expressive models of emotions in music with linear, additive musical cues, and this construction greatly decreases the complexity of any such model. Whether this holds true for other musical cues, than those studied here, remains to be verified. This also provides support for the mainly additive model that is used for combining different performance cues in the Director Musices rule system, for example, for the rendering of different emotional expressions (Bresin and Friberg, 2000).

The strength of the current approach lies in the fact that the cues and their levels can be consistently compared since the study design capitalized on a previous production study of emotional expression in music (Bresin and Friberg, 2011) and the analyses were kept comparable to past studies of expressive cues of music (Scherer and Oshinsky, 1977; Juslin and Lindström, 2010). The present study allowed us to establish plausible ranges for the cue levels in each of the manipulations. The drawback of our scheme was that the optimal sampling did not contain all the possible cue combinations. This means that the prototype examples (Figure 1) could be still be improved in terms of their emotional expression, but at least the factorial design was exhaustive enough to assess the main hypotheses about the cue level and their interactions in general. Also, our decision of using alternate sets of emotions (tender vs. peaceful) in the two laboratories was a design weakness that failed to achieve the extension of the emotions covered.

In the context of musical expression, the ranking of the importance of the musical cues for emotions seems to coalesce across the studies (e.g., Hevner, 1936; Juslin, 1997c; Gabrielsson and Lindström, 2001; Juslin and Lindström, 2010), although the small number of studies and cues studied within these studies prevents one from drawing extensive conclusions yet. We acknowledge that the choice of musical cues used for this study has, a priori, certainly excluded others from this ranking. Certain important musical cues such as *harmony, melodic contour*, or *dissonance* could be of equal relevance for attributing emotions to music and were included within the *music structure* of our design without any systematic manipulation. We also recognize that the variable contribution of the cues is a built-in feature of the brunswikian lens model, according to which communication may be accurate using multiple cues although the relative contribution of the cues will depend on the context.

As per Hevner's cautionary remarks about the results of any music and emotion study (1936), any emotional evaluations are dependent on the context established by the musical materials in question. The present work differs in three material ways from the two previous studies (Scherer and Oshinsky, 1977; Juslin, 1997c; Juslin and Lindström, 2010) that also used extensive cue manipulations. Both Scherer and Oshinsky (1977) and Juslin (1997c) used just one synthetic, artificial melody for the basis of manipulations and 2–3 large differences between the cue levels. Juslin and Lindström (2010) also had four simple melodic progressions, all based on same triadic and scalar and rhythmic elements. The present experiment was built around four polyphonic, composed and validated musical examples that were initially chosen to represent four emotion categories in a maximally clear way. Additionally, the selection of cue range was grounded in past empirical work and combined both performance-related and compositional aspects of music.

The results of the present study offer links to the findings in expressive speech research because the hypotheses about the cue direction taken from expressive speech were largely supported (Scherer, 1986; Murray and Arnott, 1993; Juslin and Laukka, 2003; Scherer, 2003). In future, it would be important to combine the factorial manipulation approach with special populations, such as children, people from different cultures, or patients with particular neural pathologies and to use other measurement techniques than self-report to further isolate the musical cues in terms of the underlying mechanisms. These combinations would allow us to determine specifically what aspects of affect perception are mostly the products of learning, as well as gain a better idea of the underlying processes involved.

### Conflict of interest statement

The authors declare that the research was conducted in the absence of any commercial or financial relationships that could be construed as a potential conflict of interest.

## Footnotes

^1^Consider a full factorial design with 8 factors, each with 2 levels (2^8^), requiring 256 combinations to be tested. For factor effects, the degrees of freedom (initially 255) would be 8 for factor *main effects*, 28 for *two-factor interaction effects* and the remaining 219 degrees of freedom (255 − 8 − 28 = 219) for the *higher order interaction* effects. In this design, 86% (219/255) of the research resources would be utilized to assess the higher-order (3rd, 4th, etc.) interaction effects that are of no primary interest and difficult to interpret. The extent of this waste of effort is proportional to the number of factor levels in the design and hence in our design, the higher order factor interactions cover 98.6% of the full factorial design matrix.

^2^More technical information about the Vienna Symphonic Library is available from (http://vsl.co.at/) and Kontakt 2 from (http://www.native-instruments.com/).

^3^http://sourceforge.net/projects/skatta/
